# The effect of equalization of public health services on the health China's migrant population: Evidence from 2018 China Migrants Dynamic Survey

**DOI:** 10.3389/fpubh.2022.1043072

**Published:** 2023-01-10

**Authors:** BinBin Su, Yu Wu, Zhao Yihao, Chen Chen, Zhong Panliang, Xiaoying Zheng

**Affiliations:** School of Population Medicine and Public Health, Chinese Academy of Medical Sciences and Peking Union Medical College, Beijing, China

**Keywords:** migrant population, equalization of public health services, health impact, China, Migrants Dynamic Survey

## Abstract

**Objectives:**

China has implemented an equalization of public health Services policy for migrants in 40 pilot cities since 2013. The main objective of this study is to explore the effect of this migrant-based reform policy on the health status of the migrant population in China.

**Methods:**

Using the China Migrants Dynamic Survey (CMDS), we included 152,000 migrants aged 15 years or over in 2018. Standardized questionnaires were used to collect socio-economic information and self-reported health status. The Associations between the equalization of public health services and health status were estimated using Multiple regression estimation models and Propensity Score Matching (PSM) methods.

**Results:**

Public health equalization reform in China has a significant and positive effect on the health status of the migrant population (β = 0.033, *p* < 0.001). Compared to males, higher income, under 60 years of age, inter-provincial mobility, and migrants those already living in urban areas, the equalization of public health Services had shown more significant positive effects on the groups who were inter-provincial migration (β = 0.055, *p* < 0.001), females (β = 0.055, *p* < 0.001), having low-income (β = 0.077, *p* < 0.001), aged over 60 years old (β = 0.191, *p* < 0.001), and living in rural areas (β = 0.038, *p* < 0.001). And multiple robustness tests prove that the above results are reliable.

**Conclusions and implications:**

Our findings confirmed the positive health effect of the equalization of public health services reform on china's migrant population, especially among vulnerable groups such as those in low income groups, in rural areas and females. And we recommend that it is necessary to further promote the practices and experiences of the pilot cities. First, strengthen health education for the mobile population and improve their health literacy. Second, further increase the financial investment to improve the coverage of public health services and the equity in resource allocation among regions. Last, strengthen the information-based management of the migrant population and prevent and control infectious diseases.

## 1. Introduction

Internal migration refers to the movement of persons within a particular geopolitical unit, which is a common phenomenon in many countries and regions worldwide, such as the United States, Europe, and China (1–5). The migrant population has made indelible contributions to China's rapid economic development, and migration is sometimes an important survival strategy for millions of people living in rural areas (6).

China has the largest migrant population in the world. According to the Seventh National Population Census, in 2020, there were 376 million migrants in China, among which 33.2% of migrants had moved from one province to another, and these figures have increased by 69.73 and 85.70% respectively, compared to the sixth National Population Census of 2010 (4). With the advancement of urbanization, the scale of the migrant population in China has changed from a continuous rising mode to a slow declining mode since 2015. The migrant population reportedly declined by 1.71 and 0.82 million respectively in 2016 and 2017 compared with 2015. Despite this, the number of migrants is still considerable due to China's huge population base.

In China, internal migrants are those whose current address does not coincide with their household registration, also known as *Hukou* (7). Most public policies and social benefits in China are configured and formulated on the basis of *Hukou* in China, rather than on the basis of the population actually living in a certain area (8). As a result, many social benefits, including health care coverage, are restricted to the urban domestic population, while the migrant population has no access or limited access (9).

While a large number of migrant movements have provided human resources to meet the growing market demand as a result of China's growing economy (10), the population health has not developed synchronously (11), and the public health services of these migrant communities lag behind that of permanent residents (12). Accompanied by the registered the residence status, the migrant population usually faces unequal treatment with the inflow of urban residents in terms of access to urban public services and social welfare, especially equal public health services (13). Therefore, they usually face more health risks than the urban residents. Although the legal right to health care for migrants varies from country to country, health practitioners report that they face similar problems when caring for the migrant population, including access problems, limited communication, and associated legal issues (14).

To improve this situation, in 2013, the Chinese government Piloted a policy for migrant populations called “Equalization Program of Basic Public Health and Family Planning Services for Migrants” (EPHSM) (15). Under this project, 40 prefectural cities were designated as pilot cities. And in these areas, a range of free policies and services to address the health needs of local internal migrants have developed. For example, establish health records, carry out health education programs and implement infectious diseases prevention measures for the migrant population. However, the health effect of this package of reform actions on the migrant population remains unclear, and there is a lack of relevant empirical studies in China. The aim of this study is to analyze the effect of EPHSM on the health of migrant populations by constructing a quasi-natural experiment on the reform of equalization of public health services for China's migrants.

This study has the following innovative contributions to the existing research gap. First, this study constructed a quasi-natural experiment to identify the effect of EPHSM on Chinese migrants' health status using the latest micro-data from China. Second, this study provides new evidence in China that public health policies can protect and improve the health status of the internal migrants. Third, the findings of this study will enable a better understanding of the public health services policy launched by the Chinese government and will ultimately be a valuable guide to interventions to improve the health among the migrant communities as well.

## 2. Materials and methods

### 2.1. Study design and data sources

China Migrants Dynamic Monitoring Survey (CMDS) is a national periodical survey carried out by the China Population Development Research Center. This nationally representative survey was first established in 2010, and then has been performed every year to investigate the migrant population in terms of the socio-economic status, health outcomes and determinations of medical services use. In this study, we used the data from the CMDS in 2018. The 2018 CMDS implemented a stratified a sampling strategy with three-stage probability proportionate sampling (PPS). 10,300 communities from 348 cities in China were included in this survey. And at the community level, 20 independent individuals were randomly selected from each community to form the final survey sample. Aged over 15 years migrants who moved across counties and lived locally for more than 1 month were interviewed. The CMDS is considered to be a good representative sample, as well as having a small sampling error (16). In total, 1,52,000 migrants were interviewed. After removing the samples with missing and extreme values of core variables, the final sample size included in this study was 1,51,639.

### 2.2. Variable measurement

#### 2.2.1. Dependent variables: The health status of the migrant population

Based on the 2018 CMDS questionnaire, this study used self-reported health to measure the overall health status of Chinese migrants. The self-rated health was indicated by choices to the question, “How do you think your health is? The options are very good, good, fair or poor?” We assigned a value of “1,” “2,” “3,” and “4” when respondents chose “very poor (unable to take care of myself),” “poor (unhealthy but able to take care of myself),” “average (basically healthy),” and “good (good healthy),” respectively. Self-reported health is considered to be a common indicator that effectively represents an individual's overall health status. Numerous studies have suggested that Self-reported health could predict the death risk and effectively represent the health level of adults (17–19).

#### 2.2.2. Independent variables: Equalization of public health services

The variable of equalization of public health services was constructed using the pilot of EPHSM carried out by the National Health Commission. The specific definition is: if the city is in the pilot list, the value is 1; If the city is not in the pilot list, the value is 0 (20).

#### 2.2.3. Controlled variables

The following variables were controlled in this analysis to minimize the possible estimation bias caused by missing variables. First, individual characteristic indicators of the migrants, including gender, age, marital status, registration local, ethnic status, political identity, family size, and flow range; Second, socio-economic status indicators, including the level of education, monthly household income, medical insurance and the types of work. Finally, to control for the effects of different urban characteristics (e.g., size, population and economic development), we also controlled city fixed effects. The specific information and descriptive analysis of the variables are shown in following (Table 1).

**Table 1:** Variable selection, definition, and assignment.

**Variable classes**	**Variable name**	**Variable definition and assignment**
Dependent variables	Self-rated health	Self-rated health: very poor = 1 (unable to take care of myself); poor = 2 (unhealthy but able to take care of myself); average = 3 (basically healthy); good = 4 (good healthy).
Independent variables	Equalization of public health services	Whether on the government pilot list: yes = 1; no = 0
Control variables	Age	Age in years
	Gender	Male = 1; Female = 0
	Marital status	Unmarried = 1; Married = 2; Divorced/widowed = 3
	Ethnic status:	Han = 1; Minority = 0
	Household registration	Rural = 1; City/town = 0
	The flow distance	Cross-provincial mobility = 1; Intra-provincial mobility = 0
	Education level	Illiteracy/elementary school = 1; Middle school = 2; High school/vocational school = 3; 3-year college = 4; 4-year college and above = 5
	Types of work	Having no work = 1; Government/state-owned enterprises/collective enterprise/the joint venture enterprise = 2; Private enterprise = 3; Individual businesses = 4; Hong Kong, Macao and Taiwan enterprises = 5; Others = 6
	Health insurance	Having no health insurance = 1; ^*^BMISURR = 2; BMISE = 3

* BMISURR, basic medical insurance scheme for urban and rural residents in China.

BMISUE, basic medical insurance scheme for employees in China.

### 2.3. Statistical methods

Stata version17.0 was used to calculate the health effect of equalization of public health services on the Chinese migrants. We adopted the OLS model, ordered probit model and ordered logit model to explore the connection between the equalization of public health services and china's migrants' health. The basic model is:


(1) $$\begin{array}{l} Health_{i}\;=\;\beta_{1}Equalization_{i}+\overset{k}{\underset{i\;=\;1}{\sum }}\beta_{k}X_{ik}+\varepsilon_{i} \end{array}$$

*Health*_*i*_ represents an individual's self-rated health status, *Equalization*_*i*_ indicates the policy of equalization of public health services. β_1_ indicates the coefficient for the effect of the reform on self-rated health, and β_*k*_ is the correlation coefficient of the control variables. ε_*i*_ is the random disturbance term. Moreover, we used the Propensity Score Matching (PSM) technology to reduce the potential endogenous problems. The PSM model is able to reduce some of the confounding effects and a number of potential endogeneity problems in the model (21). In this study, we used kernel matching, K-nearest neighbor matching and caliper matching methods to joint test the health effect.

## 3. Results

### 3.1. Descriptive results

The results of descriptive analysis is presented in Table 2. After removing the samples with missing and extreme values of core variables, we included 1,51,639 migrants in the final analysis. Of these, 53,704 migrants (35.36%) live in the pilot cities for equalization of public health Services, 78,092 (1.41%) were male, 1,38,838 (91.41%) were *Han*, 1,22,702 (80.78%) were married, 7,967 (5.25%) had political status, and 1,04,422 (68.75%) were from the rural areas. The average age of the migrant population in China was 37.05 ± 11.20 years. The mean value of health of the migrant population was 3.85 ± 0.42 scores, indicating that the health of this study population is at a relatively high level.

**Table 2:** The results of descriptive statistics.

**Variables**	**Definition**	**Mean (SE)/frequency (%)**	
**Continuous variables**		**Mean**	**SE**
Self-rated health		3.85	0.42
Age	Continuous variable: year	37.05	11.20
Family size	Continuous variable	3.13	1.19
Monthly household income	Continuous variable: yuan	7,704.17	8,380.36
**Categorical variables**		**Frequency**	**%**
Equalization of public health services	Yes = 1	53,704	35.36%
	No = 0	98,188	64.64%
Gender	Male = 1	78,092	51.41%
	Female = 0	73,800	48.59%
Marital status	Unmarried = 1	24,942	16.42%
	Married = 2	1,22,702	80.78%
	Divorced/widowed = 3	4,248	2.80%
Ethnic status:	Han = 1	1,38,838	91.41%
	Minority = 0	13,054	8.59%
Household registration	Rural = 1	1,04,422	68.75%
	City/town = 0	47,470	31.25%
The flow distance	Cross-provincial mobility = 1	76,769	50.54%
	Intra-provincial mobility = 0	75,123	49.46%
Education level	Illiteracy/elementary school = 1	24,350	16.03%
	Middle school = 2	64,063	22.39%
	High school/vocational school = 3	34,004	21.46%
	3-year college = 4	17,522	11.54%
	4-year college and above = 5	11,953	7.87%
Types of work	Having no work = 1	24,882	16.38%
	Government/state-owned enterprises/collective enterprise/the joint venture enterprise = 2	17,082	11.25%
	Private enterprise = 3	37,274	24.54%
	Individual businesses = 4	51,749	34.07%
	Hong Kong, Macao and Taiwan enterprises = 5	5,424	3.57%
	Others = 6	15,481	10.19%
Health insurance	Having no health insurance = 1	10,029	6.60%
	BMISURR = 2	1,03,931	68.42%
	BMISE = 3	37,932	24.97%

For the flow range, 76,769 (50.54%) migrant population were inter-provincial migrants, and 75,123 (49.46%) migrated within the province. In terms of socio-economic conditions, the average monthly household income of migrants was 7,704.17 ± 8,380.36 yuan. 89,023 (58.61%) migrants work in private or individual enterprises. And most of the migrants in China have health insurance (93.40%). It is worth noting that a considerable part of the migrant population is unemployed (24,882, 16.38%).

### 3.2. The effect of equalization of public health services on health

We used Maximum Likelihood Method (MLE) and the OLS method to estimate the effect of equalization of public health services on health among the migrant population. Columns (1)–(2) of Table 2 reported the association between the equalization of public health services and health using the ordered probit model. Column (1) showed that equalization of public health services was positively associated with health when we did not include any control variables (β = 0.113, *p* < 0.001). Column (2) indicated that the regression coefficient decreased to 0.033 after controlling for other confounding variables (*p* < 0.001).

To examine the robustness of the results, columns (3)–(6) of Table 2 reported the relationship between the equalization of public health services and health by using the ordered logit model and OLS method. The results also suggested that equalization of public health services was positively associated with the migrant population's health (*p* < 0.001), which was consistent with the data results in columns (1)–(2).

Moreover, among controlled variables, increasing age and family size were negatively associated with health (*p* < 0.001). In contrast, male, individuals married with a spouse, higher education level and longer flow distance were positively associated with health for the migrant population (*p* < 0.001). In terms of socio-economic indicators, higher household income and participation in BMISURR had a significant effect on the migrant population's health (*p* < 0.001). Relative to the migrant population who had no job, those with formal or informal work positively affected migrants' health (*p* < 0.001).

### 3.3. Heterogeneous analysis

Columns (1)–(2) of Table 3 divided the range of mobility of the migrants into two types: “cross-provincial mobility” and “Intra-provincial mobility,” and reported the effect of equalization of public health services on the health of the migrants under different flow distances. The results suggested that the reform of equalization of public health services significantly improved of the intra-provincial migrants' health (β = 0.055, *p* < 0.001), but it did not affect the cross-provincial migrant population.

**Table 3:** The results for the effect of equalization of public health services on the health of the migrant population in China.

**Variables**	**(1)**	**(2)**	**(3)**	**(4)**	**(5)**	**(6)**

	**Ordered probit model**		**Ordered logit model**		**OLS**	
Treat	0.108^***^	0.033^***^	0.184^***^	0.065^***^	0.031^***^	0.008^***^
	(0.008)	(0.009)	(0.016)	(0.018)	(0.002)	(0.002)
Age		−0.033^***^		−0.061^***^		−0.010^***^
		(0.000)		(0.001)		(0.000)
Gender		0.039^***^		0.086^***^		0.007^***^
		(0.009)		(0.018)		(0.002)
Middle school		0.175^***^		0.287^***^		0.069^***^
		(0.012)		(0.022)		(0.004)
High school/vocational school		0.192^***^		0.314^***^		0.064^***^
		(0.014)		(0.027)		(0.004)
3-year college		0.200^***^		0.331^***^		0.062^***^
		(0.019)		(0.038)		(0.005)
4-year college and above		0.163^***^		0.260^***^		0.052^***^
		(0.023)		(0.045)		(0.005)
Ethnic status		0.082^***^		0.156^***^		0.019^***^
		(0.015)		(0.028)		(0.004)
Married with a spouse		0.111^***^		0.165^***^		0.057^***^
		(0.017)		(0.034)		(0.003)
Divorced/widowed		0.033		0.032		0.006
		(0.026)		(0.051)		(0.009)
Family size		−0.028^***^		−0.057^***^		−0.003^**^
		(0.004)		(0.009)		(0.001)
Hukou		−0.011		−0.027		−0.004
		(0.010)		(0.020)		(0.002)
Medical insurance for urban and rural residents		0.049^**^		0.085^**^		0.010^*^
		(0.017)		(0.033)		(0.004)
Medical insurance for urban employees		0.019		0.037		−0.006
		(0.020)		(0.038)		(0.005)
Monthly household Income (ln)		0.188^***^		0.368^***^		0.059^***^
		(0.008)		(0.016)		(0.002)
Government/state-owned/collective/the joint venture enterprise		0.466^***^		0.820^***^		0.187^***^
		(0.017)		(0.034)		(0.005)
Private enterprise		0.505^***^		0.901^***^		0.190^***^
		(0.012)		(0.023)		(0.004)
Individual businesses		0.465^***^		0.826^***^		0.182^***^
		(0.014)		(0.026)		(0.004)
Hong Kong, Macao and Taiwan enterprises		0.429^***^		0.747^***^		0.172^***^
		(0.028)		(0.057)		(0.006)
Other		0.387^***^		0.678^***^		0.172^***^
		(0.016)		(0.029)		(0.005)
Flow distance		0.044^***^		0.087^***^		0.012^***^
		(0.009)		(0.017)		(0.002)
Observations	151,892	151,639	151,892	151,639	151,892	151,639
City effect	Controlled	Controlled	Controlled	Controlled	Controlled	Controlled

The numbers in double parentheses represent standard errors. ^*^, ^**^, and ^***^represent p < 0.05, p < 0.01, and p < 0.001, respectively. The reference variable for education level, health insurance, marital status and occupation types are “Illiterate/Elementary school”, “Having no health insurance”, “Unmarried” and “Having no work”, respectively.

Columns (3)–(4) of Table 3 reported the age differences regarding the effect of the public health services equalization reform on health of China's migrants. The regression results have shown that the reform had a more significant impact on the older migrant population (β = 0.191, *p* < 0.001) than on the non-elderly (< 60 years) migrant population.

Columns (5)–(6) of Table 3 reported the regional differences in the effect of the equalization reform of public health services on the health of the migrant population. The results suggested that the reform of equalization of public health services significantly improved the health level of the rural migrant population (β = 0.038, *p* < 0.001), but it had no impact on the urban migrant population.

Columns (7)–(8) of Table 3 reported the gender differences regarding the health effect of the equalization reform of public health services on China's migrants. The results indicated that the equalization reform effectively improved the health level of the female migrant population (β = 0.055, *p* < 0.001), but it had no impact on the male migrant population.

Columns (9)–(13) of Table 4 reported the income differences regarding the health effect of the equalization reform of public health services on China's migrants. The results indicated that the equalization reform effectively improved the health level of the low-income migrant population (β = 0.077, *p* < 0.001), but it had no effect on other income-level migrant populations.

**Table 4:** The regression results of the heterogeneous analysis.

**Variables**	**(1)**	**(2)**	**(3)**	**(4)**	**(5)**	**(6)**	**(7)**	**(8)**

	**Flow distance**		**Age difference**		**Urban-rural difference**		**Gender difference**	
	**Cross-provincial**	**Intra-provincial**	**15–59**	≥**60**	**Rural**	**Urban**	**Male**	**Female**
Treat	0.016	0.055^***^	0.022^*^	0.191^***^	0.038^***^	0.029	0.010	0.055^***^
	(0.013)	(0.013)	(0.010)	(0.032)	(0.011)	(0.016)	(0.013)	(0.013)
Control variables	Controlled	Controlled	Controlled	Controlled	Controlled	Controlled	Controlled	Controlled
Pseudo *R*^2^	0.108	0.142	0.084	0.094	0.121	0.139	0.115	0.139
Observations	76,646	74,993	145,471	6,168	104,228	47,411	77,969	73,670
**Variables**	**(9)**	**(10)**	**(11)**	**(12)**	**(13)**
	**Low income**	**Middle low** **income**	**Middle income**	**Middle high** **income**	**High income**
Treat	0.077^***^	0.028	0.034	−0.002	0.005
	(0.019)	(0.022)	(0.021)	(0.018)	(0.025)
Control variables	Controlled	Controlled	Controlled	Controlled	Controlled
Pseudo *R*^2^	0.172	0.097	0.088	0.077	0.084
Observations	35,873	26,499	29,067	40,579	19,621

^*^, and ^***^represent p < 0.05, and p < 0.001, respectively; Due to limited space, the results of controlled variables were not reported here.

## 4. Robustness test

### 4.1. Robustness test with different PSM methods

We examine the robustness of PSM by employing different matching techniques to effectively reduce the potential endogenous problem between EPHSM policy and migrants' health. We used different PSM matching techniques to verify the robustness of the results. Table 4 showed that the coefficients of K-nearest neighbor matching, caliper matching, and kernel matching respectively. And the figures were 0.0063, 0.0067, and 0.0073, these results were consistent with Table 5, indicating that the EPHSM policy significantly promoted the health status of the migrant population after overcoming the potential endogenous problems.

**Table 5:** PSM results for the effect of equalization of public health services on the health of the migrant population.

**Matching types**	**Treated**	**Controlled**	**ATT**	**S.E**	***T*-value**
K-nearest neighbor matching	3.8674	3.8611	0.0063	0.0028	2.25^*^
Caliper matching	3.8663	3.8596	0.0067	0.0027	2.48^**^
Kernel matching	3.8674	3.8601	0.0073	0.0025	2.92^**^

^*^, ^**^, and represent p < 0.05, and p < 0.01, respectively.

### 4.2. Robustness test with replacement of dependent variables

To resolve measurement errors in variables, We carried out several robustness tests by substituting the dependent variables with *year-round disease prevalence*, the corresponding question in the questionnaire is “*Have you been personally ill (injured) or unwell in the past year?*” and *year-round hospitalization*, whose corresponding question is “*Have you personally been hospitalized in the last year?*” Table 6 shows the results of robustness tests for replacing the dependent variable, and the results are consistent with the coefficients in Table 5. This suggests that our conclusions are relatively robust.

**Table 6:** Robustness test: replacement of dependent variables.

**Variables**	**(1)**	**(2)**	**(3)**

	**Ordered probit model**	**Ordered logit model**	**OLS**
Treat × year-round disease prevalence	0.078^***^	0.187^***^	0.006^***^
	(0.014)	(0.031)	(0.001)
Treat × year-round hospitalization	0.036^***^	0.068^***^	0.007^***^
	(0.009)	(0.018)	(0.002)
Control variables	Controlled	Controlled	Controlled
Observations	151,639	151,639	151,639

^***^indicate *p* < 0.001, respectively; Due to limited space, the results of controlled variables were not reported here.

## 5. Discussion

Based on large samples, 1,51,639 individuals were selected to identify the health effect of the EPHSM on the Chinese migrant population. In this study, we concentrated not only on the effect of the equalization of public health services on migrants' health status, but also on the health effects of different segments of migrant populations. As a developing country with the largest floating population in the world, this empirical evidence from China will have important practical significance.

The results of this study demonstrated that the reform of equalization of public health services has a significant positive effect on the self-report health in the Chinese migrant population, and the results obtained by different PSM methods were consistent with it. Migrant population cannot be granted equal access to public health services as urban residents, thus affecting the health level of the migrant population (22), As increasing health accessibility for mobile populations is the core purpose of EPHSM policy, one possible mechanism is that the equalization of public health services has increased access to health services (23), but further validation is needed.

We also found that the health status of migrant populations was at a relatively high level, which may be attributable to the “healthy immigration” and the “salmon bias” hypotheses (24). And according to *The Social Integration Assessment Report of China's Urban Migrant Population 2018*, the level of equalization of public health services in pilot cities has improved significantly compared with non-pilot cities (20). This is also partly a testament to the effectiveness of this policy.

From the perspective of other influencing factors, the results shown that the health status of the migrant population who were male, highly educated and had a stable job was better. Moreover, China's Han migrant population has better health status than ethnic minorities. These findings were consistent with other studies in China (25–27). At the same time, the insurance type of urban and rural residents played a significant role in promoting the health status of the migrant population, but the urban workers' insurance had no considerable health promotion effects (15), one possible reason was that urban workers' insurance accounts for a relatively smaller proportion of the floating population (28).

Further heterogeneity analysis found that compared to the groups who were the provincial migrations, males, having higher-than-average income and living in urban areas, equal access to public health services had shown more pronounced health effects to the groups who were inter-provincial migrants, females, having low-income and living in rural areas. It suggested that the practice of equalization of public health services in China had a more substantial health effect on the relatively vulnerable groups and paid more attention to fairness among different groups (29), which made significant contribution to help narrow the gap between urban and rural health outcomes to a certain extent (30). Migrant people who were 60 years old and above had more significant health benefits than those aged 15–69 years. This may be due to the health depreciation effect of age, making the average health level of the elder group lower than that of the young group, thus showing different health effects of the public health services equalization policy (31). Besides, we should pay more attention to the health of the elderly migrant population, since the proportion of elderly migrants has increased steadily due to greater mobility that allows families to migrate together (32). At the same time, compared with the inter-provincial migrant population, the health effect of the migrant population in the province was more significant. One possible reason was that immigrants in the province often had more medical security and family support (33–35).

This study also has the following limitations: First, for the reason of data accessibility, this study was unable to obtain self-reported health data for the mobile population prior to 2013. Therefore, it was not possible to further rigorously examine the causal relationship. Second, for the evaluation of health status, we have only analyzed a single self-assessed health indicator, lacking comprehensive judgment of multidimensional health indicators. Third, this study fails to provide an in-depth analysis of the mechanisms associated with the equalization of public health services affecting the health of migrant populations. In the future, more researches refer to causal design and multidimensional health measurement as well as mechanisms research need to be further explored.

## 6. Conclusions and implications

Overall, the equalization of public health services reform in China has significantly improved the overall health status of the internal migrant population, especially the vulnerable groups. In the future, the government should fully absorb the experience of the pilot project of equalization of public health services for the migrants and further promote the equalization of basic public services in other fields. Most importantly, the government need to narrow the gap between the migrant population and the urban registered residence population in terms of the public services and social welfare.

We make the following policy implications based on the findings above, First, the Chinese government should strengthen health education and improve the health literacy of the migrant population. It is necessary to carry out targeted health education for the migrant population groups with different characteristics from the aspects of a healthy lifestyle and health skills. Moreover, in addition to health education, we should start with universal education and use school education and social education to comprehensively improve the health literacy of the migrant population. Second, the government departments should further increase the financial investment to improve the coverage of public health services and the equity in resource allocation among regions. Third, the government should strengthen the information-based management of the migrant population and the prevention and control of infectious diseases. As a susceptible population to infectious diseases (36), especially since the outbreak of COVID-19, the massive flow of people has posed a great challenge to China's epidemic prevention and control system. We need to further strengthen the monitoring of infectious diseases among the migrant population, deal with the epidemic situation of infectious diseases promptly, and effectively implement policies such as free treatment of COVID-19, AIDS, tuberculosis, and other infectious diseases among the migrant population. The challenges posed by internal migrants in China require immediate as well as long-term efforts from the whole society and the relevant authorities and stakeholders to cope with (37).

## Data availability statement

The datasets presented in this study can be found in online repositories. The names of the repository/repositories and accession number(s) can be found below: https://www.chinaldrk.org.cn/wjw/.

## Author contributions

XZ and BS contributed to the conception or design of the work. BS, YW, CC, and ZY contributed to the work's acquisition, analysis, or interpretation of data. BS drafted the manuscript. XZ critically revised the manuscript. All gave final approval and agreed to be accountable for all aspects of work, ensuring integrity, and accuracy.
